# Combining spatially fractionated radiation therapy (SFRT) and immunotherapy opens new rays of hope for enhancing therapeutic ratio

**DOI:** 10.1016/j.ctro.2023.100691

**Published:** 2023-10-20

**Authors:** Qiuxia Lu, Weisi Yan, Alan Zhu, Slavisa Tubin, Waleed F. Mourad, Jun Yang

**Affiliations:** a Foshan Fosun Chancheng Hospital, P.R. China; b Baptist Health System, Lexington, KY, United States; c Junxin Precision Oncology Group, P.R. China; d Mayo Clinic Alix School of Medicine, Scottsdale, AZ, United States; e Albert Einstein Collage of Medicine New York, Center for Ion Therapy, Medaustron, Austria; f Department of Radiation Medicine Markey Cancer Center, University of Kentucky - College of Medicine, United States

**Keywords:** SFRT, RIBE, Abscopal effect, Immune checkpoint inhibitors (ICIs)

## Abstract

Spatially Fractionated Radiation Therapy (SFRT) is a form of radiotherapy that delivers a single large dose of radiation within the target volume in a heterogeneous pattern with regions of peak dosage and regions of under dosage. SFRT types can be defined by how the heterogeneous pattern of radiation is obtained. Immune checkpoint inhibitors (ICIs) have been approved for various malignant tumors and are widely used to treat patients with metastatic cancer. The efficacy of ICI monotherapy is limited due to the “cold” tumor microenvironment. Fractionated radiotherapy can achieve higher doses per fraction to the target tumor, and induce immune activation (immodulate tumor immunogenicity and microenvironment). Therefore, coupling ICI therapy and fractionated radiation therapy could significantly improve the outcome of metastatic cancer. This review focuses on both preclinical and clinical studies that use a combination of radiotherapy and ICI therapy in cancer.

## Highlights

• Spatially Fractionated Radiation Therapy (SFRT).
• Microvascular alterations.
• ‘RadScopal’ effect.
• Microbeam irradiation and showed significant abscopal effects in their bladders.
• Radiation-Induced Bystander Effect (RIBE).

## Introduction

Spatially Fractionated Radiation Therapy (SFRT) is a form of radiotherapy that delivers a single large dose of radiation within the target volume in a heterogeneous pattern with regions of peak dosage and regions of under dosageunderdosing. SFRT types can be defined by how the heterogeneous pattern of radiation is obtained. There are many ways to deliver heterogeneous radiation based on this concept. GRID therapy is the oldest form of SFRT and was introduced by Kohler in 1909 [1], [2]. This involves delivering a relatively high but heterogeneous radiation dose to the tumor through a perforated screen with blocked areas called a GRID. In the 1990s this was shown to be feasible with bulky and refractory tumors [3], [4], [5]. Other variations to this include the multileaf collimator (MLC) based GRID therapy where instead of a block, an MLC consisting of several sets of metallic leaves to shape the beam into GRID pattern when it exits the linear accelerator [7].

## Novel SFRT Techniques

The 3D LATTICE radiotherapy (LRT) [8], [9] is an extension of the GRID therapy that delivers a spatially fractionated radiotherapy in three dimensions.

Microplanar beam radiation therapy is another form of spatially fractionated radiotherapy. In Microbeam Radiation Therapy (MRT) [10], [11], the beam thickness ranges from 500 to 700 µm while in while in Minibeam Radiation Therapy (MBRT) [12], [13], [14]the thickness is of the order of 25–50 µm.

Microbeam Radiotherapy is another Novel Therapeutic Approach that has been investigated to Overcome Radioresistance and Enhance Anti-Tumour Response in Melanoma [15].

Abscopal effect refers to disappearance or reduction of tumor lesions outside of the field of irradiation [117].

A novel SBRT-based PArtial Tumor irradiation of HYpoxic clonogenic cells (SBRT-PATHY) has been developed to enhance the radiotherapy therapeutic ratio of advanced bulky tumors by sparing the peritumoral immune microenvironment and regional circulating lymphocytes. It has been shown SBRT-PATHY can mediate the bystander (BE) and abscopal effects (AE).

Hence, SBRT PATHY is another novel form of SFT that has been pioneered by Tubin et al in recent years [16], [17], [18], [19]. Tubin et al reported excellent clinical outcomes of inducing bystander and abscopal effects by exclusively targeting the hypoxic segment of the tumor with a high, single dose of SFRT.

SCART is another novel approach for induction of tumor immunogenicity that has been pioneered by [20], [21]. Yan et al describes this new treatment methodology 'Stereotactic Centralized Ablative Radiation Therapy' (SCART) for large tumors, which is based on the principles of SFRT, by using SBRT methods to deliver an ablative high radiation dose to central portion/hypoxic necrotic of target while keeping the dose to surrounding normal tissue to relatively low level.

**Radiobiological effects**: Radiobiological mechanisms of SFRT radiation action proceed through a number of processes with non-linear dose–response curves at low doses. These can be broadly classified as Non-immune and Immune effects:

**Non-immune effects:** Dose-volume effects: At its simplest, this is the relationship between the radiation doses that cause the same probability of a certain acute or late normal tissue damage and the irradiated proportion or the irradiated volume of the investigated tissue or organ [22], [23]. But the anatomical volume is not homogenous within organs. The relationship between anatomical/structural radiation damage and failure of organ function is different for different organs, and more related to organ physiology than to basic radiobiological concepts of cell survival.

DNA damage: The primary method of cell killing and damage done by radiation is based on the ability of ionizing radiation to damage DNA. This DNA damage can create irreparable base pair mismatches within the nucleus of the cell. There are two main methods in which DNA is damaged by ionizing radiation, direct and indirect damage. Direct damage occurs when the radiation interacts directly with the DNA strand. Indirect damage occurs when the radiation first interacts with the water in the nucleus of the cell creating a free radical. That free radical can then go forward and damage DNA. Indirect damage is more common in low linear energy transfer (LET) forms of radiation like photons or x-rays. Oxygen plays a role in the ability of a cell to repair DNA damage. When DNA damage occurs, Oxygen has the ability to react with the DNA strand at the location of the damage preventing the repair, one of the many cellular responses to DNA damage. While this occurrence in healthy tissue is an unfortunate reality, it plays a large role in the effectiveness of radiation therapy in tumor cells. The oxygen enhancement ratio or (OER) is a concept that proves the importance of oxygen concentration in cell kill with radiation. [24].

Microvascular alterations: Vascular, transvascular, and interstitial transport are affected by fractionated radiation, but that modulation is not consistent. Total doses above 45 Gy can damage tumor microvessels, while doses up to 40 Gy tend to have inconsistent effects on microvasculature. [25], [26], [27], [28], [29], [30], [31] Garcia-Barros et al in a recent communication have postulated that “high dose” radiation of 15 Gy causes an environment of Potential Lethal Damage that makes these cells sensitive to further doses of radiation, especially the endothelial cells of the tumor microvasculature, and this effect is the primary cause of tumor cell death.[32].

**Immune effects:** Findings from several preclinical studies using various animal models have suggested that the highly heterogeneous dose deposition achieved with SFRT is associated with a superior immunological response in tumor tissue as compared to homogenous radiation doses [33]. Tumor cell ablation from areas of peak dose is thought to discharge tumor antigen material that enables dendritic priming of T-cells. Lymphatic cells are then able to enter tumor tissue via the conserved perfusion of the low-dose areas [34] & [35].

Local effects: Radiation-induced bystander effects: the phenomenon in which non irradiated cells exhibit effects as a result of cell communication [36] and secreted soluble molecules [37] received from nearby irradiated cells. These effects could include DNA damage, chromosomal instability, mutation, and apoptosis.[38], [39], [40], [41], [42], [43], [44], [45]. GRID therapy can evoke significant bystander cytotoxic killing in “shielded” unirradiated tumor cells located nearby the high-dose radiated regions [41]. Immune-related phenomena are involved in mediating the bystander effect via the secretion of inflammatory mediators, interferons (IFNs) and appropriate chemokines, which attract T cells. Moreover, radiotherapy might enhance T-cell trafficking to primary tumors through local vascular endothelial inflammation [46].

Radiation therapy causes a type of sterile tissue injury, and radiation-induced inflammation is regulated by complex interactions among a variety of immune mediators which can durably reshape the immune response [47], [48], [49]. The cytokine profile of tumor cell lines that have been exposed to fractional radiation is different from acute radiation [50]. Radiation therapy can bring about cell death through different mechanisms like mitotic catastrophe, apoptosis, necrosis, autophagy and senescence. The extent of the curative role of these processes is likely to be cell, tissue, tumor and radiation dose-dependent.

Radiation can modify the response of the immune system by immune stimulation or immunosuppression [51], [52], [53], [54], [55], [56], [57], [58], [59], [60], [61], [62]. SFRT induces a greater-immunomodulatory gene expression than regular radiation therapy [55], [59], [63], [64], [65], [67], [68], [69].

Abscopal effects and bystander effects are hypothesized [66]to be immune- or cytokine-based. Spatially-fractionated radiation therapy approaches have been shown to induce these effects [16], [70], [30], [20]. Stereotactic Body RadioTherapy (SBRT)-based PArtial Tumor irradiation targeting HYpoxic segment (SBRT-PATHY) is a model of partial tumor irradiation purposefully developed to spare the peritumoral immune-microenvironment from radiation and in doing so it induces immune-mediated bystander effects and abscopal effects [18].

**Immune suppressive effects:** Radiation therapy can induce various immune suppressive effects.[71], [72], [73], [74]. It kills irradiated effector immune cells and upregulates immunosuppressive molecules in the tumor microenvironment. Clinical data show that the conventional radiotherapy predominantly generates immune-suppressive effects [70].

**Fig. 1:**
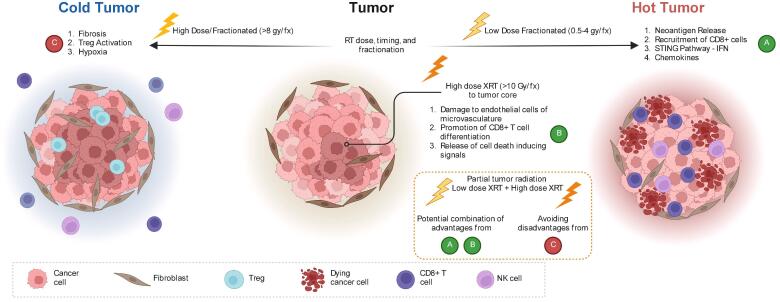
**Schematic overview of local effects triggered by partial tumor irradiation.** At the heart of the primary tumor that is irradiated with high dose (in the middle), three effects can be distinguished: endothelial cells death and vasculature damage; induction of CD8 T cells and cell death signals release. Current evidence suggests that high dose radiation per fraction can induce immunosuppression on tumor edge while low dose radiation can induce immunomodulatory effect via neoantigen release, recruitment of CD8+ T cells to tumor edge and activation of IFN pathway genes. As a result, partial tumor radiation is primed to attack tumors with high dose in the core and achieve a low dose radiation to tumor edge with rapid dose fall off. This strategy would potentially combine the inherent advantages of various fractionation schemes while avoiding the potential pitfalls of immunosuppressive effects rendered by high dose radiation to the whole tumor.

**Immune stimulatory effects:** Radiation therapy can also evoke immune stimulatory effects [18], [34], [72], [76], [77]. Radiation therapy can induce dendritic cell maturation and antigen release from tumors [75], [46], [79], [80]. It can enhance priming of antigen-specific T cells [78], [81], [82], and increase -T cell trafficking and infiltration [83], [84]. CD8 + T cell proliferation [82], [85], [86], [87], [88] and tumor immunogenicity [89], [90], [91] can be enhanced by radiation therapy. This brings about immunogenic cell death [74], [92], [93], [94] (Fig. 1).

Distant effect: The ‘RadScopal’ effect arises as a consequence of radiation directed to part of the primary tumor, and may be accompanied by low-dose RT towards secondary lesions. It has been proposed that this double form of radiation, combined with ICIs, might be able to modulate the TME of both primary and secondary tumors, maximizing the anti-tumor immune responses [95], [96]. Barsoumian et al. investigated the priming of T cells in the Lewis lung carcinoma (LLC) mouse model using high-dose RT to a higher tumor burden. They further investigated the combination of low-dose RT to the metastatic sites, in order to modulate the stroma. Interestingly low-dose RT enhanced the anti-tumor responses to ICIs (anti-PD-1 and anti-CTLA-4), promoting M1-polarization of macrophages, NK infiltration, and reduction in transforming growth factor beta (TGF-β) levels. These data suggest that low-dose RT can reprogram the TME, improve the infiltration and function of effector immune cells in the secondary tumors, and are able to overcome the inhibitory effects of the tumor stroma. Finally, the combination with the two ICIs could further increase and prolong the systemic effects of RT, via T regs blocking and attenuation of T cell exhaustion [95], [96].

**Anti-Tumor Immunity Elicited by SFRT in Pre-clinical Studies**: The radiation scheme of SFRT results in the administration of ablative- and low-dose irradiation in different regions. In conventional radiation therapy the combination of a single, high ablative dose followed by subsequent lower doses can convert the immunosuppressive microenvironment to a more immunogenic one with increased infiltration of immune effector cells and a downregulation of regulatory T cells [94]. To the authors’ best knowledge, detailed studies comparing differences in immune responses between SFRT and conventional RT have not been performed yet in patients. Studies on C57BL/6 mice implanted with Lewis lung carcinoma 1 cells and then treated with a three-dimensional volume-based lattice radiation therapy (LRT) have shown that it can elicit both local and metastatic/distant tumor control through modulation of the tumor immune microenvironment. It increased secretion of inflammatory cytokines and elevated immune cell infiltration compared to un-irradiated tumors [98]. This shows that high-dose partial volume irradiation can cause an improved distant effect than the effect induced by total tumor volume irradiation through activating the host immune system.

Microbeam radiation therapy (MRT) has been compared against broad beam radiation therapy (BBRT) in murine mammary carcinoma models. MRT treated mice had a general decrease in tumor-associated-macrophages (TAMs) while the BBRT group had increased TAMs relative to the un-irradiated controls[63]. EMT6.5 tumors that received MRT vs BBRT differentially regulated immunity-related genes [59], [65]. An antitumor immune response was evoked by Partial-Volume Single-Dose Radiation in 67NR murine orthotopic breast tumors [64]. There are other reviews[96]that have extensively discussed the combinations of immunotherapy and local radiotherapy in preclinical tumor models.

Athymic nude mice injected with F98 glioma cells into their right cerebral hemisphere were exposed to pencil beam or microbeam irradiation and showed significant abscopal effects in their bladders [97]. Murine tumor models have demonstrated that partial tumor irradiation is responsible for an increased immune-mediated tumor cell death in unirradiated distant tumors compared to the whole tumor irradiation [98]. Despite this preclinical evidence of abscopal effects, it remains controversial in clinical settings [66]. Interestingly, when the exodus of CD8 + T cells from LNs was inhibited, there were no effects on tumor response, suggesting that the source of infiltrating T lymphocytes were not the DLNs, but most likely the targeted section of the hemi-irradiated tumor. Overall, this study suggests that partial irradiation alone can be immune-stimulatory and indirectly control tumor growth via immune activation, inducing an abscopal effect in the contralateral tumor. Preclinical data with LRT radiation in mice model showed that LRT can elicit both local and metastatic/distant tumor control [99].

The Radiation Induced Abscopal Effect (RIAE) might be due to modulation of the tumor immune environment, or through triggering systemic immune responses. Single-fraction spatially fractionated radiation therapy (SFRT) significantly delayed unirradiated distant tumor growth in a mice Xenograft lung tumor model. LRT induced increased secretion of inflammatory cytokines in mice serum. Interestingly, infiltration of CD3 + T cells increased significantly in the right-sided unirradiated tumor after irradiation with LRT to 50 % tumor volume in the left-sided leg tumor. This finding suggested that cellular immunity might play a role in LRT-triggered AR. Furthermore, What is more interesting was that high-dose partial volume irradiation with LRT resulted in increased CD3 T-cell infiltration in the unirradiated tumor, compared with whole tumor volume irradiation. Thus, partial tumor irradiation might activate the host immune system in a different manner compared with whole tumor volume irradiation [100].

Fractionated but not single-dose radiotherapy has been shown to induce an immune-mediated abscopal effect when combined with anti-CTLA-4 antibodies in TSA mouse breast carcinoma and MCA38 mouse colon carcinoma models [101]. Immunocompetent mouse models with a triple negative breast tumor (4T1) when treated with GRID and antibodies against immune checkpoints PD1 and CTLA-4 showed that a systemic immune activation to a primary tumor, can promote anti-tumor immune responses outside the radiation field [102]. These studies show that combined radio-immunotherapy where the radiation therapy is spatially fractionated could augment both local and metastatic disease responses. In the next section we will explore clinical studies that combine SFRT and immune-therapies together to get better cancer treatment.

**Anti-Tumor Immunity Elicited by SFRT in Clinical Studies:** Hypofractionated radiotherapy (HFRT) can raise inflammatory cytokine levels more than compared to conventional fractionated radiotherapy [103]. When combined with immune checkpoint blockade, a higher tumor control effect was observed in HFRT treated mice compared to those treated with conventional radiotherapy schemes [104]. There is a case report of combining of High-Dose LATTICE radiation therapy with immune checkpoint blockade in a patient with non-small cell lung cancer with multiple metastases. The metastatic lesion that received both spatially fractionated radiation therapy combined with anti-PD1 immunotherapy showed complete local response five months after treatment [35]. Stereotactic body radiotherapy on a single tumor site preceding pembrolizumab treatment has been shown to enhance overall response rate in patients with advanced non-small cell lung cancer [106]. In a clinical trial, a fractionated radiation scheme combined with pembrolizumab showed safe antitumor activity in patients with advanced solid tumors [105]. In a case study [6], a patient presented with metastatic melanoma and was refractory to pembrolizumab (anti-PD1). The patient received spatially fractionated GRID radiation therapy with pembrolizumab for five months, and his neck mass was completely resolved. In a prospective single-institution trial (NCT02303990) pembrolizumab was tested in combination with HFRT in patients with metastatic cancers (NSCLC, melanoma, pancreas, breast, others). This showed that combining anti-PD1 with HFRT was well tolerated by patients and led to prolonged and complete response despite previous progression on anti-PD-1 therapy alone [108]. In another clinical study (NCT02383212) patients with advanced solid tumors were treated with cemiplimab (anti-PD-1). When this was combined with HFRT, it showed an augmented response suggesting abscopal effects [106]. In contrast to these studies, a randomized phase I/II trial showed that pembrolizumab when concurrently given with SBRT or HFRT showed no benefits in median progression-free survival or overall survival when compared with pembrolizumab without radiation therapy [107]. This suggests that larger trials are required to address which patients benefit most from combining SBRT/HFRT with immune checkpoint blockade, or a different strategy with HFRT is needed. It has been hypothesized that proteins are able to withstand freezing and thawing, might be responsible for transmitting the bystander signal from irradiated to naïve bystander cells [111], [112], [101], [36], [37], [39], [40], [41], [42], [43], [44], [45], [16], [17], [18], [19]. Reactive oxygen species, growth factors, and cytokines have been implicated in the maintenance of the bystander signal. Their Our results suggest that secreted factors that lead to reactive oxygen species are a very likely candidate for the effects observed in vitro, since the authors we observed the greatest increase in expression of antioxidant genes immediately following GRID treatment. One mechanism for Radiation-Induced Bystander Effect (RIBE) might be through radiation-induced cytokines. Indeed, TNF-α and TRAIL are directly involved in apoptosis and have been suggested to play a role in bystander effects. These are induced in cells that are under the high-dose open GRID areas. Plus, increased therapeutic efficacy of GRID therapy is associated with TNF-α induction in the serum obtained from patients treated [113], [114], [115], [116], [16], [17], [18], [19].

Mohiuddin et al. [6] reported a case of a locally advanced melanoma tumor that initially responded to ipilimumab, but then progressed and became refractory to multiple systemic, immunological agents, including ipilimumab, high-dose IL-2, and pembrolizumab. The patient's tumor was then introduced to high-dose radiation along with the anti-PD-1 agent, pembrolizumab, and the patient had a rapid, complete response to treatment in the neck with minimal side effects. The patient’s tumor had previously progressed on five cycles of pembrolizumab alone prior to radiation. Conventional radiation of 50 Gy alone would not generate the dramatic, rapid response seen here, either. That case is important because it suggests that high-dose radiation can be used as an immunological primer for re-introducing sensitivity to biological agents.

Formenti and Demaria [99] suggested that the local radiation on the patient’s tissue acts as an in-situ vaccine. Radiation targeted to the intact primary tumor can release radiation-specific antigens and induce attracting chemokines to activated T-cells, thus engaging the patient’s innate immune response against the tumor. If the tumor-specific immune response is strong enough, it can also enable systemic regression in areas outside of the local radiation field, known as the abscopal effect.

In SFRT using microbeam therapy, GRID or LATTICE in animal models, a strong bystander effect has been shown in unirradiated tumor cells adjacent to cells exposed to high dose radiation [41]. This is not seen in conventional radiotherapy. It is likely that these abscopal effects in SFRT are due to immunogenic pathways. Indeed, in clinical studies partial bulky-tumor irradiation shows abscopal effects in patients [19]. SBRT-PATHY is a fractionated radiation therapy developed for unresectable bulky tumors and it exploits radiation-hypoxia-induced non-targeted effects like bystander effects and the abscopal effects [17]. In a clinical trial “metabolism-guided” lattice radiotherapy using radiation dosages that produce bystander, abscopal, and immunological effects yielded a clinical response of 89 %, including 23 % of complete remission in stage IV bulky tumor disease patients [118]. Preclinical studies suggest that these bystander effects are mediated by cytokines [109], such as TRAIL [111] and TNF [116].

Since the immune system plays an important role as the mediator/effector of RIBEs/RIAEs, SBRT-PATHY planning considers peritumoral immune microenvironment and the circulating regional lymphocytes as the new organ at risk that should be spared by radiation and ‘protected’ by its own dose constraints. In this published study, the bulky tumor control rate (RIBE response rate) among patients with unresectable NSCLC treated exclusively with SBRT-PATHY was 95 %. RIAEs were seen in 45 % of patients. The authors reported on possible relationship between RIBE and RIAE, stating that a significantly higher AR rate has been seen in patients in which more intense RIBE occurred (i.e., partially treated tumors reduced for >50 %). SBRT-PATHY concept implied that for successful therapeutic immune modulation, the entire tumor volume may not need to be irradiated. This should initiate more optimal antigen shedding, increase effector T-cell activation, and lead to favorable alterations in radiation-spared peritumoral immune microenvironment. Recently, an Italian group also confirmed the efficacy of SBRT-PATHY in their initial experience. Results from preclinical mouse models from Memorial Sloan Kettering Cancer Center also support the hypothesis behind SBRT-PATHY. The authors investigated tumor response to partial irradiation in 67 NR murine orthotopic breast tumors in both immunocompetent and nude mice. Partial tumor irradiation with a single dose of 10 Gy delivered to half of the tumor led to reproducibly inducible antitumor immune responses that eliminates the entire tumor in immunocompetent mice, but not in nude mice. In addition, a significant RIAE was observed after partial irradiation with a single dose of 10 Gy. The results of this study were comparable to those previously achieved in the clinic by using SBRT-PATHY [16], [17], [18], [19].

The studies highlighted in this section show that spatially fractionated radiation renews or enhances an immunological response: The timing for combining radiotherapy and immune checkpoint inhibition therapy could also affect the response from of the combination therapy. Preclinical studies on this are not conclusive about the optimal timing from with studies that suggesting concurrent therapy is better [119] to studies that show no significant difference [120] in treatment outcomes based on sequential or concurrent delivery of the two treatment modalities. The issue of timing is also not well resolved in clinical studies. Several clinical trials suggest that patients receiving immune checkpoint inhibitors immediately after radiotherapy might have better clinical outcomes [113], [110], but there are also clinical trials that show that immune checkpoint inhibition prior to SBRT has better outcomes [115], or that there are no significant differences in sequential vs concurrent treatment [121]. These studies suggest that optimization of the combined combining radiotherapy and immune checkpoint inhibition therapy is likely to be dependent on the type of cancer, number of lesions, and kind of fractionated radiotherapy being used.

SFRT and Immunomodulation/Immunotherapy in Clinical Studies: Conventional radiotherapy and fractionated radiotherapy by itself can function as an immunomodulator [9], [122]. In prostate cancer patients, significant CD68, and CD163 macrophages increase and decrease respectively in CD8 T cells has been observed within two weeks following prostate Stereotactic Body Radiotherapy (SBRT). Differences in radiotherapy fractionation can result in distinct immune-modulatory effects [120], so future clinical studies could test different radiation therapy regimens to see how radiation itself functions as an immunomodulator.

We have presented evidence in the previous sections for immune activation with high dose radiation and for preclinical studies where various SFRT is combined with immunotherapy. Other reviews [114], [121], [123], [124], [125], [126], [127] have described in detail the synergism and potential clinical implications of combining immune checkpoint inhibitors with radiotherapy. It is therefore the logical leap to consider that SFRT coupled with immune checkpoint blockade would be effective and could subsequently lead to improved local tumor control without added toxicities.). A case report describes the case of a patient treated with a novel form of immune-sparing partially ablative irradiation (ISPART) for a bulky peritoneal metastasis from renal cell cancer, refractory to anti-PD-1 therapy (nivolumab) as third-line therapy after sequential therapy with sunitinib and cabozantinib. The observed response suggests that there may be a synergistic effect between ISPART and immunotherapy. This case report supports the inclusion of ISPART in patients presenting with bulky lesions treated with checkpoint inhibitors [128].

**Summary:** SFRT has the potential to overcome tumor radio resistance and modulate the local immune response, which could lead to significant improvement in tumor control locally, regionally, and distantly while preserving healthy tissues. Combining this with immune checkpoint inhibitors is a promising strategy for treating metastatic cancers and can trigger abscopal effects. We have discussed here the anti-tumor immunity elicited by fractionated radiotherapy and its role in immunomodulation. Wide acceptance of this combinatorial treatment will require further research to optimize the fractional radiotherapy and immune checkpoint therapy parameters that would allow for maximizing an immune response while still reducing the adverse effects of radiotherapy. It is reasonable to hypothesize that radiation could modulate immune functions in its applications such as dose, fractionation size, tumor site and size, and location of nearby organs at risk.

If the hypothesis is proved, radiomics and genomics studies would be valuable to elucidate the effect of radiation in human body in a systematic manner. On occasions, it is difficult or impractical to deliver the higher dose per fraction ideal for eliciting an anti-tumor immune response due to tumor size or location. Under these circumstances, RT may be delivered by irradiating a fractional tumor volume. Spatially fractionated radiation therapy (SFRT) is a way to deliver radiation to a whole or partial tumor with inhomogeneous radiation dose. We suspect that anti-PD-1/anti-PD-L1 combination with SFRT will be translated into meaningful Phase II and Phase III trials and lead to improvement in outcomes for metastatic patients. Knowledge of radiotherapy causing increased antigen shedding and increased expression of neoantigens paved the pathway for combination approaches with anti-CTLA-4. Similarly, advances in checkpoint agonists and cytokines may also provide additional avenues of combination trials with radiotherapy. Future trials should incorporate these immunotherapy agents with modern radiotherapy and use a more biologically targeted, organs at risk sparing approach, which may include the *peri*-tumor microenvironment.

## Declaration of Competing Interest

The authors declare that they have no known competing financial interests or personal relationships that could have appeared to influence the work reported in this paper.
